# The development and evaluation of a quality assessment framework for reuse of dietary intake data: an FNS-Cloud study

**DOI:** 10.3389/fnut.2025.1519401

**Published:** 2025-06-06

**Authors:** Laura A. Bardon, Grace Bennett, Michelle Weech, Faustina Hwang, Eve F. A. Kelly, Julie A. Lovegrove, Panče Panov, Siân Astley, Paul Finglas, Eileen R. Gibney

**Affiliations:** ^1^Food and Nutrition National Bioscience Research Infrastructure, Quadram Institute Bioscience, Norwich, United Kingdom; ^2^Institute of Food and Health, University College Dublin (UCD), Dublin, Ireland; ^3^School of Agriculture and Food Science, University College Dublin, Dublin, Ireland; ^4^Department of Food and Nutritional Science, Hugh Sinclair Unit of Human Nutrition, Institute of Food, Nutrition and Health, University of Reading, Reading, United Kingdom; ^5^Department of Food and Nutritional Science, Hugh Sinclair Unit of Human Nutrition, Institute for Cardiovascular and Metabolic Research, University of Reading, Reading, United Kingdom; ^6^Biomedical Engineering Section, School of Biological Sciences, University of Reading, Reading, United Kingdom; ^7^Department of Knowledge Technologies, Jožef Stefan Institute, Ljubljana, Slovenia; ^8^EuroFIR AISBL, Brussels, Belgium

**Keywords:** data reuse, dietary intake data, quality assessment, tool development, lifestyle data, demographic data

## Abstract

A key aim of the FNS-Cloud project (grant agreement no. 863059) was to overcome fragmentation within food, nutrition and health data through development of tools and services facilitating matching and merging of data to promote increased reuse. However, in an era of increasing data reuse, it is imperative that the scientific quality of data analysis is maintained. Whilst it is true that many datasets *can* be reused, questions remain regarding whether they *should* be, thus, there is a need to support researchers making such a decision. This paper describes the development and evaluation of the FNS-Cloud data quality assessment tool for dietary intake datasets. Markers of quality were identified from the literature for dietary intake, lifestyle, demographic, anthropometric, and consumer behavior data at all levels of data generation (data collection, underlying data sources used, dataset management and data analysis). These markers informed the development of a quality assessment framework, which comprised of decision trees and feedback messages relating to each quality parameter. These fed into a report provided to the researcher on completion of the assessment, with considerations to support them in deciding whether the dataset is appropriate for reuse. This quality assessment framework was transformed into an online tool and a user evaluation study undertaken. Participants recruited from three centres (*N* = 13) were observed and interviewed while using the tool to assess the quality of a dataset they were familiar with. Participants positively rated the assessment format and feedback messages in helping them assess the quality of a dataset. Several participants quoted the tool as being potentially useful in training students and inexperienced researchers in the use of secondary datasets. This quality assessment tool, deployed within FNS-Cloud, is openly accessible to users as one of the first steps in identifying datasets suitable for use in their specific analyses. It is intended to support researchers in their decision-making process of whether previously collected datasets under consideration for reuse are fit their new intended research purposes. While it has been developed and evaluated, further testing and refinement of this resource would improve its applicability to a broader range of users.

## Introduction

Within the field of nutrition research, there is a wealth of existing dietary intake datasets that have been collected within national, regional or targeted sub-population group studies. Fewer studies exist that have been collected across multiple countries or regions. The few pan-European nutritional studies that do exist, including the Food4Me study (1), Feel4Diabetes study (2), EPIC (3) and the Seven Countries study (4), enable deeper analyses to be completed including country-to-country comparisons. Although these analyses are invaluable in nutrition research, they are costly representing a loss of scientific opportunities and waste of time and financial resources. Data reuse and merging of existing datasets can help achieve insights without the same time and expenses but strategies to effectively reuse data need to be considered.

Numerous methodological approaches to the collection and analysis of data exist, making it challenging to merge or compare datasets (5). In more recent years, initiatives such as EUMenu by EFSA have sought to create harmonised data collection approaches across countries facilitating comparison of different datasets or merging of datasets for combined analysis (6). Furthermore, initiatives including FAO/WHO GIFT (7) and the Global Dietary Database (8) are examples of how datasets can be harmonised and accessed for effective reuse by the community. Currently, large amounts of (often publicly funded) money are used to generate big datasets that are usually not exploited for reuse, despite this increasingly becoming a requirement for funding bodies.

FAIR principles (findable, accessible, interoperable and reusable) were established as a guide to support maximal benefits from data, tools, services and algorithms (9). Applying FAIR principles to data is mutually beneficial for both scientific research and society. Recognising this, the European Commission (EC) has established an expert group that aims to turn the concept into reality to open up science and research (10), through the European Open Science Cloud (EOSC), which federates existing European research infrastructures and aims to realise a web of FAIR data and related services, making more data interoperable and machine actionable (11). Making data FAIR is an increasingly important requirement of European funding requirements and is likely to be mandatory in future (with some exceptions), enabling existing datasets to be accessed and reused. These principles were applied in Food Nutrition Security Cloud (FNS-Cloud) (grant agreement no. 863059) underpinning data reuse (9). FNS-Cloud aimed to improve access to datasets, tools and services in the domains of food, nutrition and health, making access more equable across Europe enhancing research capacity through defragmentation of food, nutrition, and security data and the development of tools and services to facilitate matching and merging of data to promote increased data reuse (12).

In this era of increasing data reuse, when using existing, open datasets to answer new research questions, it is important that researchers understand and consider the quality and provenance of data before being reused (13, 14). Challenges exist around dietary intake data due to the variety of methods for collection available, approaches to describe/quantify portion sizes, and underlying composition tables used to generate mean daily intakes; these should be adequately considered before reusing dietary intake data. Several dietary assessment methods exist to collect dietary data at food group or individual food item levels, including food frequency questionnaires or 24-h dietary recalls and food diaries (15, 16). Depending on the method chosen, portion size of foods can be quantified (using actual weights) or estimated (including using portion size pictures, household measures, photographic food atlases or by applying average intakes). There are many food composition datasets available. These can be national composition tables, such as the Composition of Foods Integrated Database (CoFID) for the United Kingdom, or databases for larger regions, such as the EFSA database for Europe (17). Selection of a composition dataset, which is appropriate for the population examined, is essential to ensure the accuracy of resulting data. These challenges, among others, impact the accuracy of resulting data and how it can be used. Dietary intake data has a range of uses including development of food based dietary guidelines, assessment of nutrient deficiencies in populations, and examination of dietary, meal patterns, and food choice in a given population or subgroup (18, 19).

Although development of a prototype Cloud infrastructure through the FNS-Cloud project represents an advancement, and a new direction for food and nutrition science, it is important that data are reused appropriately, to ensure the quality of resulting scientific outputs remain high. When considering the quality of specific datasets, it is important to note this must be in the context of an individual research question. Each user must assess whether the datasets they have selected are appropriate for their research question. This relies on scientific integrity among researchers and appropriate knowledge of datasets prior to reuse. Whilst the onus is, and should remain, on researchers to ensure outputs are based on sound science, there is also a need to support researchers in the decision-making process of whether a dataset is appropriate for their purpose. Within FNS-Cloud, a quality assessment tool acts as a guide for data users to assess whether a dietary intake dataset is suitable to answer their research question, facilitating an informed final decision by the data user. Thus, the aim of this work was to develop and evaluate a quality assessment framework for FNS-Cloud to support researchers in their decision-making process around data reuse, specifically if datasets under consideration are *fit for their intended purpose*.

## Methods

Development of this framework followed processes for developing any quality assessment tool, as described by Whiting et al. (20). This approach consisted of three stages, initial steps (defining scope, identification of parameters of quality), tool development (development of dietary intake dataset quality assessment decision trees, output from decision trees, testing of framework design, transformation of quality assessment framework into an online tool, evaluation of quality assessment tool and contents) and dissemination. An overview of the actions taken within this body of work is summarised in Table 1.

**Table 1:** Overview of the process of developing the quality assessment framework.

Stage	Quality assessment framework for dietary intake, consumer behavior, lifestyle and demographic data for FNS-Cloud
Stage 1: Initial steps	
1.1 Identify need	A tool to support the reuse of existing dietary intake datasets
1.2 Obtain funding	This work was conducted within Food Nutrition Security Cloud (FNS-Cloud) (grant agreement no. 863059)
1.3 Assemble team	Larger group FNS-Cloud Consortium (*n* = 35 partner institutions) Working group (*n* = 15 researchers across 7 partner institutions)
1.4 Manage project	Core group (UCD, *n* = 3 researchers)
1.5 Define scope	Appropriateness of reuse of existing dietary intake datasets Domain based flowcharts Questions with defined answer options and personalised messages with considerations
Stage 2: Tool development	
2.1. Generate items	Targeted literature review of data domains focusing on data collection, data handling, use of underlying data sources, data uses and analysis Summarised parameters of quality Formation of trees
2.2. Agree items	Virtual face-to-face meeting
2.3. Produce first draft	Core group
2.4. Pilot and refine	(1) Paper based feedback from consortium members on main data domain (dietary intake data) (2) Application of paper-based version of the form on *n* = 19 datasets across 2 example research questions (3) External feedback through evaluation activity in 3 centres
Stage 3: Dissemination	
3.1 Publication	Planned peer review publication of tool development process Entry of tool into FNS Cloud catalogues
3.2 Website	Integration into FNS-Cloud (https://catalogues.fns.foodcase-services.com/catalogues)
3.3 Uptake	Presentation to FNS-Cloud consortium
3.4 Translations	None planned thus far

### Defining scope

As described, the aim was to develop a quality assessment framework and user-friendly tool to support selection of dietary intake data for reuse, thereby ensuring the quality of outputs is maintained when exploiting data in future research. The core domain of interest for this framework was dietary intake data, however additional FNS-Cloud data domains—including demographic, anthropometric, lifestyle and consumer behavior data—were also included as they are often collected in conjunction with dietary intake data for context. Inclusion of multiple data domains expands the types of research questions that can be answered and, therefore, maximises the scope of food and nutrition data that might be included. For example, links can be made between lifestyle, diet and the development of health conditions; and the food environment can impact consumer behavior and subsequent dietary intake. Other complementary data collected generally encompasses lifestyle, physical activity, and measures of consumer behavior such as purchase, preparation, and consumption.

### Identification of parameters of quality

Firstly, to identify parameters of quality, targeted searches of peer-reviewed literature (including PubMed Central and Web of Science) were performed for each of the domains (dietary intake, lifestyle, anthropometric, demographic, consumer behavior). Searches focused on where quality can be affected during data generation, namely during collection (method of collection chosen, validation, period of collection, days of week, training of data collectors), selection of underlying data sources (portion size quantification, composition databases), how raw data were handled (identification of under/over-reporters, systems used for coding foods), and uses and analysis of data (whether analysing data based on nutrients, foods or food groups). From this review, individual parameters of quality were identified; these were reviewed by researchers with expert knowledge where additional or overlooked parameters of quality were identified.

### Development of dietary intake dataset quality assessment decision tree

Once the parameters were defined, assessment was developed in the form of decision trees. An overview of the structure of the decision trees is presented in Supplementary Figures 1A–C. Decision trees have been used previously in healthcare to support clinical decision making (21, 22) and also in the delivery of personalised nutrition advice (23). The parameters of quality were transformed into questions with structured categorical answer options, e.g., yes, no, do not know. Follow up questions were developed, where necessary, forming branches within the decision tree. Individual branches were developed for each data domain with separate branches also created at different levels where quality can be impacted in the generation of dietary intake data. Each branch concluded with delivery of a personalised message based on the answers selected. The personalised messages give information on the parameter in question, describing why the parameter is important and how it might influence quality of the dataset based on the answer(s) selected.

### Output from decision trees

Following completion of the quality assessment, a user is presented with a personalised feedback report compiling all messages that were produced. The content of these messages varies depending on the relevance to parameters in question but provide the user with considerations to support their decision on whether to use the dataset to answer their research question. Key findings from the literature review of quality parameters for the data domains informed the content of these feedback messages.

### Testing of framework design

A prototype decision tree framework consisting of decision trees illustrated in a powerpoint format was developed and tested in two phases. First, internal testing was conducted at a face-to-face workshop during an FNS-Cloud consortium meeting, attended by ~30 food and health researchers from across the FNS-Cloud partner institutions (*n* = 35 institutions across 12 EU member states, Serbia and Switzerland) in Sardinia, Italy in June 2022, whereby the structure of the dietary intake data branches (questions, response options, and messages) were presented and feedback collected. The consortium comprised a diverse group of nutrition researchers, IT professionals, software developers, and communications specialists. Participating consortium members were asked to review the framework contents and evaluate whether any parameters of quality were missing; the appropriateness of the questions and responses; and, whether the messages were useful for the researcher. Their feedback was used to modify the prototype and develop complementary data branches of the framework. Once fully developed, the paper-based framework was used in case studies by two independent researchers (LAB, MW) to manually assess the quality of existing datasets that had previously been identified to answer two (example) research questions [*N* = 11 datasets assessed for research question 1: “*What are the factors influencing dietary patterns and adherence to sustainable healthy eating guidelines across Europe?”* (LAB) and *N* = 8 datasets assessed for research question 2: *“Does diet quality and dietary intake differ across key adult life stages, and are these influenced by demographic factors, such as European region and sex?”* (MW)]. Researchers answered each question in the framework and created a table of feedback responses the tool generated for each dataset. An informed decision regarding whether each dataset was suitable for reuse to answer the research question was made considering the feedback messages received.

### Transformation of quality assessment framework into online tool

**Figure 1:**
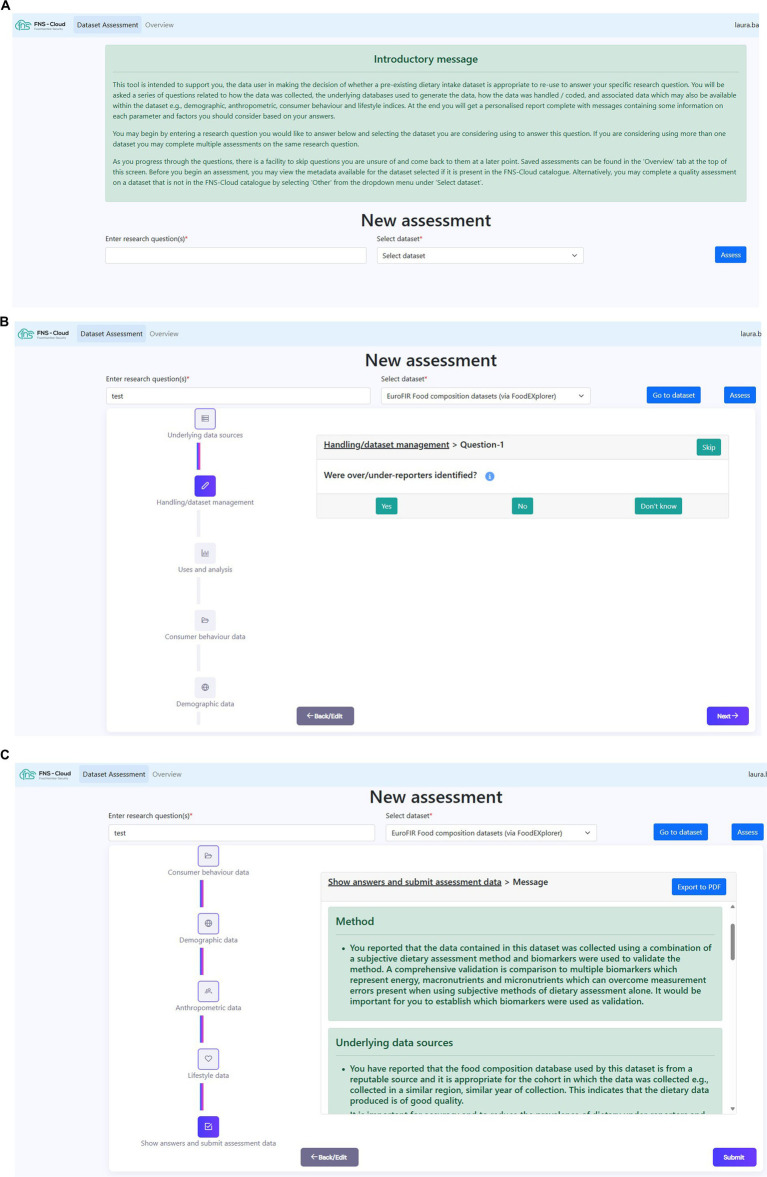
Snapshots of developed quality assessment tool. **(A)** Tool introductory page. **(B)** Dataset assessment flow. **(C)** Personalised feedback report.

Following feedback and testing, the revised decision trees were transformed from paper-based format into conditional expressions (IF/THEN statements) and a prototype of the online dietary intake data quality assessment tool produced (Figures 1A–C). Then followed an iterative refinement process between two researchers from the core development group (LAB and GB) who identified issues, bugs and glitches in the prototype and the technical team^1^ who solved the identified problems. Example research questions were formulated and used to test the accuracy of the workflow. Suggestions for improving the usability of the tool were also shared with the technical team.

### Critical evaluation of the tool and its contents

Following development of the online tool, wider evaluation was conducted among a group of participants with prior research experience in analysing dietary intake data. These evaluations were performed either virtually using Microsoft Teams or in person and were conducted from August 2023 until January 2024 at three centres: University College Dublin (UCD), Ireland (researchers GB, LAB); University of Reading (UoR), United Kingdom (researchers FH, MW); and Quadram Institute Bioscience (QIB), Norwich, United Kingdom (researcher LAB). The researchers undertook targeted recruitment of people with dietary intake domain knowledge within their departments so that they could evaluate the appropriateness of the tool contents and feedback messages. Ethical approval was granted by the UoR research ethics committee (number: 32/2023) and informed written consent was obtained from each participant before interviews were conducted.

Participants were asked to use the tool to perform a quality assessment on a dietary intake dataset that they had prior experience of using. Participants were provided with the URL to the tool and login details, after which they completed independently. Researchers observed them to determine how users navigated the tool and collected comments from participants as they were using the tool. Following completion, these participants were interviewed using an indicative interview script co-developed and agreed previously by all researchers. This guide included a list of questions to ask the participants to assess critical elements of the tool, quality assessment, and feedback messages. All interviews (virtual and in-person) were recorded, with the consent of participants, for later analysis. Basic demographic information including participant sex, career stage, years of experience with dietary intake data, and, self-rated experience and knowledge of dietary intake data were captured through multiple choice questions. Finally, participants were shown and asked a number of open-ended and Likert-item questions to (1) evaluate clarity of the tool’s purpose and whether users could navigate the tool easily, (2) verify the tool’s contents and assess the relevance and clarity of questions, and (3) gather overall feedback on the tool and its future usability.

To analyse user evaluations of the tool, automated transcripts of the interview videos were generated using Microsoft Teams and subsequently verified using the recordings. One researcher (LAB) reviewed all interview recordings and transcripts multiple times to extract content as well as observational data from interviewer notes. Data were collated for each participant individually, under section headings used during the assessment (for example specific response and reaction to tool content and use of question hints). An inductive analytical approach was applied whereby key phrases discussed by participants were identified (24). An inductive analytical approach allows the content of data to inform emerging patterns. Similar statements of feedback were collated and assigned category labels to determine the frequency with which certain opinions were mentioned by participants. Researchers applied a code frequency approach to determine key themes. All identified categories of feedback were divided into overarching themes of positive elements of the tool or elements requiring future consideration. Participant feedback on the assessment, feedback messages, and overall tool experience was reviewed by two researchers (LAB and GB).

## Results

### Quality framework development

An overview of the parameters of quality identified within each data domain is presented in Supplementary Table 1. *N* = 25 parameters of quality were identified with the majority (60%) being within the dietary intake data domain. Individual decision trees were developed for demographic, dietary intake, consumer behavior, anthropometric and lifestyle data. The dietary intake decision trees contained four levels: “data collection,” “data handling/dataset management,” “underlying data sources applied,” and “uses and analysis.” Lifestyle data was divided into “data collection” and “data handling/dataset management,” and consisted of *n* = 3 branches, *n* = 4 distinct questions and *n* = 5 distinct personalised messages. The remaining three data domains (consumer behavior, demographic and anthropometric data) had one branch each with a total of *n* = 4, *n* = 5 and *n* = 3 distinct questions, and *n* = 8, *n* = 4 and *n* = 5 distinct messages, respectively. All feedback messages developed for the decision trees are presented in Supplementary Table 2. Under uses and analysis, a decision was made not to create a decision tree asking about parameters of quality due to the wide range of analytical possibilities. Instead, a generic message was produced describing considerations when using and analysing dietary intake data. Dietary intake data was predominant with *n* = 7 branches, *n* = 24 distinct questions and *n* = 37 distinct personalised messages. All domains were divided into levels where quality can be affected during generation of data. Table 2 provides a breakdown of the branches within each data domain, and the numbers of distinct questions and messages developed for each.

**Table 2:** Overview of numbers of branches, questions and messages within the tool.

Data domain	Branches	Branches (*n*)	Distinct questions (*n*)	Distinct messages (*n*)
Dietary intake	Methods	7	12	16
	Underlying data sources A		8	14
	Underlying data sources B			
	Underlying data sources C			
	Underlying data sources D			
	Handling of data A		4	7
	Handling of data B			
Consumer behavior	Methods	1	4	6
Demographic	Methods	1	5	8
Anthropometric	Methods	1	3	4
Lifestyle	Methods A	3	4	5
	Methods B			
	Methods C			

Following creation of the online prototype of the tool (Figures 1A–C), modifications included addition of an introductory message, describing the purpose of the tool for users; user ability to skip questions; user ability to save completed assessments; pop-up help icons to further explain certain terminology within the questions; and, ability to download personalised feedback report after the assessment.

### Evaluation of the tool and contents

A total of *n* = 13 participants (*n* = 5 UCD, *n* = 5 UoR, *n* = 3 QIB) completed the evaluation; the average interview time was 1 h and 9 min and ten participants completed the evaluation virtually via Microsoft Teams. Responses to structured demographic questions are shown in Table 3. Most respondents were female (77%), had been working with dietary intake data for at least four years (69%), and considered themselves to be very experienced with dietary intake data (62%). Participants were from a range of career stages but almost half (46%) were postgraduate students.

**Table 3:** Demographic characteristics of evaluation study participants.

	*N* (%)
Female sex	10 (76.9)
Education/career stage	
Postgraduate student	6 (46.2)
Postdoctoral researcher	3 (23.1)
Researcher <5 years	0
Researcher 5–9 years	2 (15.4)
Researcher >10y years	2 (15.4)
Years experience with dietary intake data	
<1 year	0
1–3 years	4 (30.8)
4–6 years	4 (30.8)
>6 years	5 (38.5)
Self-rated experience with dietary intake data	
Moderately experienced	5 (38.5)
Very experienced	8 (61.5)
Extremely experienced	0
Self-rated knowledge of dietary intake data quality	
Moderately knowledgeable	7 (53.8)
Very knowledgeable	5 (38.5)
Extremely knowledgeable	1 (7.7)

**Figure 2:**
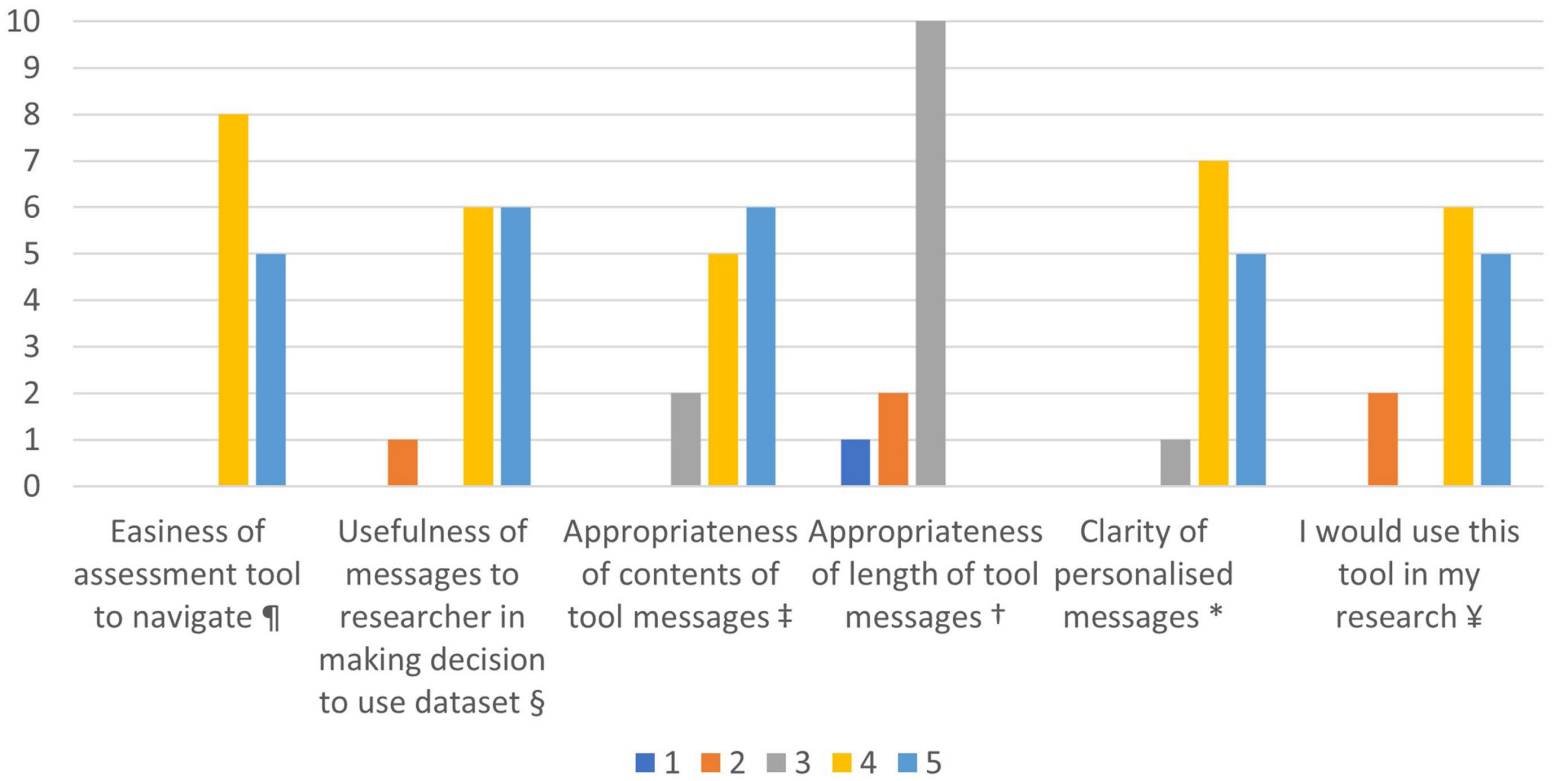
Participant self-ratings of aspects of the tool. ¥ 1 = strongly disagree, 2 = somewhat disagree, 3 = neutral, 4 = somewhat agree, 5 = strongly agree. * 1 = very unclear, 2 = unclear, 3 = neutral, 4 = clear, 5 = very clear. † 1 = way too long, 2 = too long, 3 = about right, 4 = too short, 5 = way too short. ‡ 1 = not at all appropriate, 2 = somewhat inappropriate, 3 = neutral, 4 = somewhat appropriate, 5 = very appropriate. § 1 = being useless, 2 = somewhat useless, 3 = neutral, 4 = somewhat useful, 5 = very useful. ¶ 1 = very difficult, 2 = somewhat difficult, 3 = neither difficult nor easy, 4 = somewhat easy, 5 = very easy.

Overall, participants rated individual aspects of the tool positively (Figure 2). All participants rated the assessment format as either “somewhat easy” or “very easy” to navigate (*n* = 13, 100%), and the majority felt the information in the personalised feedback report was “somewhat useful” (*n* = 6, 46%) or “very useful” (*n* = 6, 46%) in helping decide if a dataset was appropriate to reuse for their purpose. When rating the messages, the majority rated the contents as “somewhat” or “very appropriate” (*n* = 11, 85%), length as “about right” (*n* = 10, 77%), and the clarity as “clear” or “very clear” (*n* = 12, 92%). All except two participants “agreed” or “strongly agreed” that they would use the tool in their research. The majority of participants (*n* = 8, 62%) rated the user friendliness of the tool as “excellent”.

Feedback from the user evaluation was categorized into positive aspects and facets that needed future consideration (Table 4). In general, participants were positive about the tool and its contents. Some believed they would use this tool for future research (*n* = 10, 77%), primarily with datasets they have not collected themselves (*n* = 2, 15%) as this would help identify the strengths and weaknesses of a dataset. Even those who did not feel the tool would be useful for their work, did speak about benefits for students or inexperienced researchers (*n* = 5, 38%). The information asked for during the assessment, especially within the dietary intake data section, was deemed relevant for measuring quality of data and included some information that is often poorly considered in dietary research. However, some elements of the tool were described as text heavy, in particular the tool introduction page and the feedback messages for lifestyle and demographic data domains. In addition, feedback messages were not always deemed useful for specific research purposes nor were they based on specific responses provided during the assessment, being described as overly generic. Some specific improvements such as altering the wording of some questions within the assessment as well as specific technical and functional improvements were suggested by participants (Supplementary Table 3).

**Table 4:** User feedback from evaluation study.

Aspect	Positive aspects identified	Aspects in need of future consideration
Tool	Tool purpose was well understood (*n* = 8, 62%). Useful for inexperienced users of nutrition data (*n* = 8, 62%) or during study design phase (*n* = 4, 31%).	Introductory message – text heavy (*n* = 4, 31%). Consider how research question is incorporated into assessment (*n* = 2, 15%). Ideally data owner would complete assessment; challenging and time consuming for users unfamiliar with the data (*n* = 8, 62%). Technical improvements such as a side panel listing questions to display progress (*n* = 4, 31%).
Assessment	Overall questions were deemed as important and relevant to assessing data quality (*n* = 12, 92%). Hints associated with each question were appreciated and used throughout assessment (*n* = 10, 77%).	Phrasing could be improved for certain questions/data elements that all users may not be familiar with, e.g., food coding systems (*n* = 6, 46%). A greater number of response options or the option to select multiple responses would be useful (*n* = 8, 62%). Some additional questions were suggested, listed in Supplementary material (*n* = 5, 38%).
Feedback report	Dietary intake and anthropometric data domain reports were clear, easily understood and examples were appropriate (*n* = 7, 54%). Information included in dietary intake feedback report was deemed relevant to quality of nutrition data (*n* = 5, 38%).	Feedback provided was overly generic. Messaging could be tailored to the specific dataset/research question provided (*n* = 5, 38%). Lifestyle and demographic messages were repetitive (*n* = 8, 62%). Report sections were quite long and wordy (*n* = 4, 31%). Consider visual presentation of information (*n* = 2, 15%). Would like definitive indication of usability or good/bad quality rating (*n* = 2, 15%).

*n*=: indicates the number of participants who discussed these sentiments in their assessment of their tool.

## Discussion

This paper presents the development and user evaluation of a novel quality assessment tool for dietary intake data designed for use in nutrition research. The tool was designed to assess appropriateness of existing dietary intake datasets for reuse in addressing new research questions. User evaluation was undertaken to understand potential applicability and functionality of the tool. The tool was intended for use within nutrition research with the user evaluation identifying inexperienced nutrition researchers and students as ideal users.

As research questions around nutrition are increasingly focused on food security, sustainable diets, and the interplay of diets with health and environmental consequences, effective nutrition research increasingly requires data from multiple disciplines. In the absence of largescale multiple country databases with data from many areas, there is a greater need for merging datasets for secondary uses. Data reuse and exploitation for new aims presents many opportunities to improve the pace of research and increase capacity to answer more complex problems facing society. However, as part of researcher integrity, user communities have a duty to ensure scientific quality is not compromised. The development of tools and frameworks are an important part of this transition to facilitate data reuse and ensure that researchers are adequately supported. This tool was designed to act as a support for the researcher, but responsibility still lies with the researcher to ensure they adequately understand the dataset in question before deciding to use it. Furthermore, supporting data reuse underpinned by FAIR principles are priorities for European Open Science Cloud (*EOSC*) (11). To ensure these are successfully implemented in the health and life sciences communities, there is a need to upskill researchers and to engage data curators. This was emphasized in the user evaluation, where over half the participants suggested tool assessment would be quicker and feedback possibly more accurate if data owner(s)/provider(s) completed the assessment.

Study participants spoke about the importance of supporting users to assess the appropriateness of reusing dietary intake data. While most felt the duration of the assessment was appropriate, some were concerned about the time it might take new users to complete, who were not familiar with the selected dataset. These participants believed that, ideally, the owner/provider of the data should complete the quality assessment of their dataset, as they would have greater knowledge about the methodologies used. This would revise the scope of the tool, whereby potential users are presented with a report about the strengths and weaknesses of the dataset, and under which circumstances the dataset might be appropriate to be used in. Additional aspects of data quality such as questions about the size and age range of the population, representativeness of the sample, measurement of anthropometrics in fasted vs. unfasted participants, and seasonal variation in intakes were deemed missing from the tool, both in the assessment and feedback report, which many participants expressed as important when assessing quality of food and nutrition data. Guidelines on dietary assessment have been developed in a similar way, highlighting the importance of an open and reiterative process, refining the contents following a series of expert panel reviews (25).

Whilst there are several quality assessment initiatives and tools that have been developed for the food and nutrition domain (26) such as Nutritools (25, 27), Quisper,^2^ and DAPA^3^, to the best of the authors’ knowledge, this is the first attempt to assess the quality of previously collected data for reuse. Like other quality assessment frameworks, the design of this tool is not intended to definitively advise the user whether datasets are suitable to answer research questions; rather, the tool supports decision making through personalised messages containing additional quality and ‘fitness for use’ factors they may not have previously considered. Quality assessment frameworks are not designed to recommend a single best approach. Instead, they provide a systematic approach to ascertain whether a certain element is fit for the intended purpose and provide suggestions on how to approach different situations (20). Within the space of medical research, several frameworks have been developed in an attempt to systematically assess the quality of health records for reuse (28–31). Some of these frameworks have since been expanded and tailored for specific areas of research, such as heart failure biomarkers to promote identification of appropriate quality studies for reuse in this field (32). The work presented in this paper takes a more generalized approach, as the framework can be applied to all types of dietary intake data but goes beyond previous frameworks as it has been transformed into an online open access tool that can be easily accessed and used by all.

### Future work

This paper presents the development of a quality assessment framework for assessing dietary intake datasets for reuse and its transformation into a first iteration tool. Although a user evaluation study showed the tool was broadly accepted and a particular value was seen in training inexperienced researchers and students in thinking about data quality, the tool would benefit from further development to optimize the user experience. The tool could be further developed to be formally included in nutrition sciences curricula as a training resource for students. Several participants cited the desire for a definitive rating of the datasets quality thus there is a need to make the purpose of the tool, to support the researcher’s own decision making, clearer. Participant feedback has highlighted revisions that would be useful to include in a next version of the software, mainly around the need to condense text on the introductory page and in certain feedback messages as well as addition of further response options and hint icons (user support). The current text heavy version of the tool may be unappealing and a barrier to use for some users who deem it too time consuming. In order to improve the tool’s uptake, some participants suggested visualisation of results or generating a summary table of “key messages.” Large amounts of text could mean that users less experienced with dietary data may misinterpret or become overwhelmed by the information provided. Furthermore, the evaluation study described in this paper included only thirteen participants, predominantly postgraduate students, who were recruited from 3 research centres across the United Kingdom and Ireland. This may limit the generalizability of our findings to other groups outside of these locations thus a broader evaluation study including more diverse participants from other geographical locations would be important so that it could be used more broadly. Evaluation of the tool by a wider variety of intended users (research, clinical, non-nutrition disciplines) alongside a wider range of experience levels may identify additional improvements which could be made to the tools content and clarity. It is intended to be a living tool that can be further developed and potentially expanded over time. Participants in the user evaluation suggested tailoring some questions and or responses to the research question provided. Addressing these elements could be the focus of any future iterations. Within this first iteration, one data type (dietary intake data) was chosen and the most commonly associated sub-data types (demographic, lifestyle, anthropometric, consumer behavior) added. This list is not exhaustive and future versions could be expanded to include further data types.

### Strengths and limitations

There are many strengths to the tool. Quality parameters were identified through a combination of literature searches and knowledge from domain experts. The tool utilizes a standardized framework that asks consistent questions and covers all areas where quality might be affected during data generation—from data collection, data handling processes, use of underlying data sources, through to how the data are intended to be used and analysed. To the authors’ knowledge, no such tool currently exists for dietary intake datasets thus it addresses an important gap. Further, the tool has potential to enhance research capacity through supporting researchers to address new research questions by exploiting existing data.

Some limitations must also be acknowledged. Although the design and tool have been evaluated by test users, there could still be relevant parameters that have not been identified or included. Almost half of the participants in the user evaluation were postgraduate students and there was a lack of participants with extensive experience which may have impacted the findings. User evaluation interviews were conducted by three separate sets of researchers across three different centres as opposed to a single researcher. To minimize differences emerging as a result of this, researchers co-developed a single interview script that was followed for all interviews.

## Conclusion

The tool presented here can support users assessing the suitability of dietary intake datasets for reuse. Although not designed to definitively inform a user whether a dataset is appropriate for their purpose, the use of personalised feedback messages provides users with important considerations to support decision-making. In particular, evaluation of the tool suggested that students and early career researchers might benefit most and the tool could have benefits as a training resource to develop their thinking. The tool is openly available from the FNS-Cloud platform.^4^ Future work could expand this framework to incorporate further data types.

## Data Availability

The original contributions presented in the study are included in the article/Supplementary material, further inquiries can be directed to the corresponding author.

## Footnotes

^1^ www.scalefocus.com

^2^ https://quisper.eu/

^3^ https://www.measurement-toolkit.org/

^4^ https://aai.fnscloud.eu/realms/fns-cloud/protocol/openid-connect/auth?client_id=sf-da-public-stag&redirect_uri=https%3A%2F%2Fdataset-assessment.scalefocus.dev%2F&state=a9d16e40-eef1-4036-ae84-1d5eb7c70e0d&response_mode=fragment&response_type=code&scope=openid&nonce=b1a5e35c-6259-4394-8375-7c10b19f7b0f
