# Supplementary material for: The development and evaluation of a quality assessment framework for reuse of dietary intake data: an FNS-Cloud study
Source: Front Nutr. 2025 Jun 6;12:1519401. doi: 10.3389/fnut.2025.1519401 (PMC12178894; doi:10.3389/fnut.2025.1519401)
Supplement: Supplementary file 1 [file Table_1.docx]

**Supplementary Table 1. Parameters of quality identified within each data domain**

|  | **Dietary Intake data** | **Anthropometric data** | **Demographic data** | **Lifestyle data** | **Consumer behaviour data** |
| --- | --- | --- | --- | --- | --- |
| **Data collection** | - Method chosen - Tool validation - Researcher training - Length and timing of data collection | - Method chosen - Training provided | - Method chosen - Questionnaire validation - Researcher training - Terminology used - Response rates | - Method chosen - Questionnaire validation - Terminology used - Response rates | - Method chosen - Questionnaire validation - Population considered |
| **Data handling / dataset management** | - Source of food composition database - Portion size quantification - Completeness of nutrient information - Number of days of data collection - Identification of mis-reporters | None | None | - Units used - Identification of mis-reporters | None |
| **Underlying data sources applied** | - Coding system used for classifying foods | None | None | None | None |
| **Uses and analysis** | - Nutrients - Individual foods / food groups - Dietary patterns - Meal patterns - Risk assessment | None | None | None | None |

| **Supplementary Table 2. Individual messages developed for each data domain** | |
| --- | --- |
| **Dietary intake data domain** | |
|  | *Methods* |
| M1.1 | You reported that the tool used to collect this data is not validated. It is important that any tool used in dietary intake assessment is validated before use. Data arising from non-validated tools should be used with caution. It may be better to use data that has been derived from a validated tool. |
| M1.2 | You reported that the tool used to collect this data has been validated by comparing it against an objective biomarker(s). A comprehensive validation is comparison to multiple biomarkers which represent energy, macronutrients and micronutrients. It would be important for you to seek further information to fully understand how validity was determined in this tool. |
| M1.3 | You reported that the tool used to collect this data was validated by comparing it to another method of dietary intake data collection. It would be important for you to seek further information on the validation study(s) conducted to understand which method the tool was compared against and how valid the tool was that was considered. |
| M1.4 | You reported that the tool used to collect this data was validated by comparing it to an objective biomarker(s) and another method of dietary intake data collection. It would be important for you to fully understand what method(s) of dietary intake data collection were considered and what biomarkers were considered and the relative validity across these. |
| M1.5 | You reported that the study population in this dataset is similar to the population in which the tool was validated and that the validation study population was an appropriate size. When using a validated dietary intake assessment tool it is important that the study population in which the tool was validated is similar to the population considered in this dataset. It is always important that tools are used in similar populations to that in which they are validated. For example, if the tool has been validated for use in children it may not be appropriate for use in adult populations. Caution should be taken if the number of people included in the validation study(s) is very small. |
| M1.6 | You reported that the study population in this dataset is either not similar to the population in which the tool was validated or that the population used in the validation study was not an appropriate size. When using a validated tool it is important that the study population in which the tool was validated is similar to the population considered in this dataset. It is always important that tools are used in similar populations to that in which they are validated. For example, if the tool has been validated for use in children it may not be appropriate for use in adult populations. |
| M1.7 | You reported that you are unsure whether the FFQ used to collect this data was validated or not. It would be important for you to find this information out as all tools used in dietary intake assessment should be validated before use. Data arising from non-validated tools should be used with caution. |
| M1.8 | You reported that the data contained in this dataset was collected using an interviewer-led 24-hour recall(s) which were conducted by trained individuals. Evidence has demonstrated that when the interviewers conducting 24-hour recalls are trained the precision and accuracy of the data collected is improved. |
| M1.9 | You reported that the data contained in this dataset was collected using an interviewer-led 24-hour recall(s) which were conducted by untrained individuals. Evidence has demonstrated that when the interviewers conducting 24-hour recalls are trained the precision and accuracy of the data collected is improved. Details on the methods used to collect this data are lacking and it would be important for you to find out more information before using this dataset. |
| M1.10 | You reported that the data contained in this dataset was collected using paper-based food diaries where the participants were provided with instructions on how to accurately complete them. Provision of instructions for participants can improve the detail and quality of the dietary information provided. The researcher should establish how this paper-based data was then entered into the database. Use of standardised instructions and coding rules with skilled researchers when entering data into the database can improve the accuracy of the dietary data produced. |
| M1.11 | You reported that the data contained in this dataset was collected using paper-based food diaries where the participants were not provided with instructions on how to accurately complete them. Provision of instructions for participants can improve the detail and quality of information provided. The researcher should also establish how this paper-based data was then entered into the database. Use of standardised instructions and coding rules with skilled researchers when entering data into the database can improve the accuracy of the dietary data produced. |
| M1.12 | You reported that the data contained in this dataset was collected using an alternative subjective method of dietary data collection such as image recognition, barcode scanning, retail/shopping data or using an app. You should consider whether this novel method of dietary intake data collection has been validated, whether it is appropriate for the population group within this dataset and the underlying data sources (e.g., nutrient composition database) used to generate the dietary data. |
| M1.13 | You reported that the data contained in this dataset was collected using a combination of a subjective dietary assessment method and biomarkers were used to validate the method. A comprehensive validation is comparison to multiple biomarkers which represent energy, macronutrients and micronutrients which can overcome measurement errors present when using subjective methods of dietary assessment alone. It would be important for you to establish which biomarkers were used as validation. |
| M1.14 | You reported that the data contained in this dataset was collected using concentration biomarkers such as plasma vitamin C or carotenoids. Concentration biomarkers are associated with dietary intake and used to rank individuals’ intakes. |
| M1.15 | You reported that the data contained in this dataset was collected using predictive biomarkers such as urinary sucrose or fructose. It is important to understand that whilst predictive biomarkers do not completely reflect dietary intakes they can predict them. |
| M1.16 | You reported that the data contained in this dataset was collected using recovery biomarkers such as doubly labelled water, urinary nitrogen or potassium. Recovery biomarkers are directly associated with dietary intake and can be used to assess absolute intakes. |
|  | *Underlying data sources A* |
| M2.1 | You have reported that the source of the food composition database used by this dataset is either unknown or not named. When using dietary intake data it is important to understand where the underlying food composition data comes from. Food composition databases have been shown to be one of the most influential determinants in comparing energy and nutrient intakes among different commercial apps. This is particularly important if a researcher is considering using data arising from a technology-based method, as these are often ‘user determined’ or a combination of user derived data and published datasets, thus making it difficult to determine the quality of the nutrient intake data produced. |
| M2.2 | You have reported that the food composition database used by this dataset is not from a reputable source. Food composition databases have been shown to be one of the most influential determinants in comparing energy and nutrient intakes among different commercial apps. This makes it difficult to determine the quality of the nutrient intake data produced. |
| M2.3 | You have reported that the source of the food composition database used by this dataset is known yet you are unsure whether it is a reputable source. When using dietary intake data it is important to understand where the underlying food composition data comes from. Food composition databases have been shown to be one of the most influential determinants in comparing energy and nutrient intakes among different commercial apps. This is particularly important if a researcher is considering using data arising from a technology-based method, as these are often ‘user determined’ or a combination of user derived data and published datasets. You should find out more information about the composition database used in order to determine the quality of the nutrient intake data produced. |
| M2.4 | You have reported that the food composition database used by this dataset is from a reputable source and it is appropriate for the cohort in which the data was collected e.g., collected in a similar region, similar year of collection. This indicates that the dietary data produced is of good quality. |
| M2.5 | You have reported that the food composition database used by this dataset is from a reputable source but that it is not appropriate for the cohort in which this dataset was collected e.g., collected in a similar region, similar year of collection. Food composition databases have been shown to be one of the most influential determinants in comparing energy and nutrient intakes among different commercial apps. Therefore, the researcher should consider how different the composition database used is to their cohort in order to determine the quality of the dietary data produced. |
|  | *Underlying data sources B* |
| M2.6 | It is important for accuracy and to reduce the prevalence of dietary under-reporters and over-reporters that portion size is accurately quantified in dietary intake datasets. You reported that portion size was quantified in this dataset using estimations. This may have been done using photos / household measures / average weights from existing data. Estimating portion size may increase the prevalence of dietary under-reporters. Online tools incorporating images of multiple portion sizes may reduce this error, however these methods rely on participant memory so may not be appropriate for all population groups. |
| M2.12 | It is important for accuracy and to reduce the prevalence of dietary under-reporters and over-reporters that portion size is accurately quantified in dietary intake datasets. You reported that portion size was quantified in this dataset using a combination of measured and estimated weights. The estimated measures may have been quantified using photos / household measures / average weights from existing data. Estimating portion size may increase the prevalence of dietary under-reporters. Online tools incorporating images of multiple portion sizes may reduce this error, however these methods rely on participant memory so may not be appropriate for all population groups. |
| M2.13 | It is important for accuracy and to reduce the prevalence of dietary under-reporters and over-reporters that portion size is accurately quantified in dietary intake datasets. You reported that portion size was quantified in this dataset using measured weights. |
| M2.14 | It is important for accuracy and to reduce the prevalence of dietary under-reporters and over-reporters that portion size is accurately quantified in dietary intake datasets. You reported that this dataset did not quantify portion size however the data handlers may have applied average portion sizes to the data. It would be important to determine where these average portion sizes came from and whether they are appropriate for the study population in order to determine the quality of the data produced. |
| M2.15 | You reported that you are unsure whether portion sizes were quantified in this dataset. It would be important for you to find out this information before using this dataset as accurate quantification of portion size in dietary intake datasets is necessary for accuracy and to reduce the prevalence of dietary under-reporters and over-reporters. |
|  | *Underlying data sources C* |
| M2.7 | You reported that this dataset contains nutrient information for a wide selection of macronutrients and micronutrients. This dataset would be appropriate for answering a wide range of research questions focusing on contribution of foods/food groups to nutrient intakes, adherence to nutrient guidelines, dietary patterns and diet quality. |
| M2.8 | You reported that this dataset only contains nutrient information for calories and macronutrients or a limited number of micronutrients. The data is appropriate for research questions focusing on analysis at a food level, or questions assessing food choice and preferences in a given population. |
| M2.16 | You reported that you do not know the extent of the nutrient information provided within this dataset. It would be important for you to find this information out before deciding whether to use the dataset as the amount of nutrient information affects the types of research questions the data can be used to answer. For example, datasets containing a wide selection of macronutrients and micronutrients are appropriate for answering a wide range of research questions focusing on contribution of foods/food groups to nutrient intakes, adherence to nutrient guidelines, dietary patterns and diet quality whilst datasets which only contain nutrient information for calories and macronutrients are appropriate for answering research questions focusing on analysis at a food level, or questions assessing food choice and preferences in a given population. |
|  | *Underlying data sources D* |
| M2.9 | You reported that this dataset collected dietary information on multiple days and included both weekdays and weekend days. Collection of data across multiple days is suggested to increase the overall data quality, where nutrient intakes can be calculated as a daily average, giving a more accurate representation of an individual’ diet. Furthermore, an individual’s diet often varies between weekdays and weekend days thus many studies aim to collect data across the week, to account for this individual variation. |
| M2.10 | You reported that this dataset collected dietary information on multiple days. Collection of data across multiple days is suggested to increase the overall data quality, where nutrient intakes can be calculated as a daily average, giving a more accurate representation of an individual’s overall diet. However, this dataset did not capture information for both weekday and weekend day intakes. As an individual’s diet often varies between weekdays and weekend days, many studies aim to collect across the week, to account for this individual variation. |
| M2.11 | You reported that this dataset collected dietary information for individuals on a single day. Collection of data across multiple days is suggested to increase the overall data quality, where nutrient intakes can be calculated as a daily average, giving a more accurate representation of an individual’s overall diet. Furthermore, individual’s diets often vary between weekdays and weekend days, thus capturing dietary information across week and weekend days can capture this individual variation. |
| M2.17 | You reported that this dataset collected dietary information on multiple days yet you do not know whether data was collected across both week and weekend days in this dataset. Collection of data across multiple days is suggested to increase the overall data quality, where nutrient intakes can be calculated as a daily average, giving a more accurate representation of an individual’ diet. Furthermore, an individual’s diet often varies between weekdays and weekend days thus many studies aim to collect data across the week, to account for this individual variation. It would be important for you to find out more information about this dataset before deciding whether or not to use it. |
|  | *Data handling / dataset management A* |
| M3.1 | You reported that inadequate dietary reporters (dietary under-reporters and dietary over-reporters) were identified in this dataset and that sensitivity analysis (completion of analysis both including and excluding inadequate dietary reporters) was previously completed to assess the impact of inadequate reporters on the study results. This is important to assess the accuracy of the data collected. Depending on the prevalence of inadequate reporters and the impact (if any) of these inadequate reporters on the overall study results, the researcher may consider excluding them from the analysis. |
| M3.2 | You reported that inadequate dietary reporters (dietary under-reporters and dietary over-reporters) were identified within the dataset, however sensitivity analysis (completion of analysis both including and excluding inadequate dietary reporters) to understand the effect of inadequate dietary reporters on the study results was not completed. Depending on the prevalence of inadequate reporters, you may want to consider completing sensitivity analysis when using this dataset. |
| M3.3 | You reported that this dataset did not identify people who inadequately reported their diet (by either over or under quantifying their intake). Identification of inadequate dietary reporters is important to understand how accurate the data is. Comparison of reported energy intake to Basal metabolic rate (BMR) and physical activity level is frequently used to identify inadequate reporters and understand the prevalence within a given study population. Sensitivity analysis (completion of analysis both including and excluding inadequate dietary reporters) can then be undertaken to assess the impact of inadequate reporters on the results. The researcher should consider identifying inadequate dietary reporters when using this dataset. |
| M3.8 | You reported that you do not know whether inadequate dietary reporters (dietary under-reporters and dietary over-reporters) were identified in this dataset. It would be important for you to find out this information before deciding to use the data as identification of inadequate dietary reporters is important to understand how accurate the data is. Comparison of reported energy intake to Basal metabolic rate (BMR) and physical activity level is frequently used to identify inadequate reporters and understand the prevalence within a given study population. You may want to investigate the prevalence of inadequate reporters and consider completing sensitivity analysis (completion of analysis both including and excluding inadequate dietary reporters) when using this dataset. |
| M3.9 | You reported that inadequate dietary reporters (dietary under-reporters and dietary over-reporters) were identified within the dataset, however you do not know whether sensitivity analysis (completion of analysis both including and excluding inadequate dietary reporters) to understand the effect of inadequate dietary reporters on the study results was completed. Depending on the prevalence of inadequate reporters, you may want to consider completing sensitivity analysis when using this dataset. |
|  | *Data handling / dataset management B* |
| M3.4 | You reported that the food data included in this dataset has been coded using a standardised coding system such as FoodEx2 or LanguaL. The accuracy of dietary intake data is highly dependent on the detail and precision used by researchers in coding the data and the appropriateness of the underlying food composition database. Use of a standardised coding system increases the possibilities for merging of this dataset with other dietary intake datasets for completion of harmonised analyses. |
| M3.5 | You reported that the food data included in this dataset has been coded using a detailed food coding system which may be specific to the country in which the data was collected. The accuracy of dietary intake data is highly dependent on the detail and precision used by researchers in coding the data and the appropriateness of the underlying food composition database. |
| M3.6 | You reported that the food data included in this dataset has not been coded using detailed food descriptions or a standardised food coding system. The accuracy of dietary intake data is highly dependent on the detail and precision used by researchers in coding the data and the appropriateness of the underlying food composition database. The researcher should consider if the level of detail included in this dataset is appropriate to answer their research question before selecting this dataset for use. |
| M3.7 | You reported that you do not know whether the food data included in this dataset has been coded using detailed food descriptions. It is important for you to understand the level of detail included in this dataset as the accuracy of dietary intake data is highly dependent on the detail and precision used by researchers in coding the data and the appropriateness of the underlying food composition database. The researcher should consider if the level of detail included in this dataset is appropriate to answer their research question before selecting this dataset for use. |
| M3.8 | You reported that you do not know whether the food data included in this dataset has been coded using a standardised coding system such as FoodEx2 or LanguaL. You should find this information out before using this dataset as the accuracy of dietary intake data is highly dependent on the detail and precision used by researchers in coding the data and the appropriateness of the underlying food composition database. Use of a standardised coding system increases the possibilities for merging of this dataset with other dietary intake datasets for completion of harmonised analyses. |
|  | *Uses and analysis* |
| **Generic M** | Dietary intake data varies depending on the method used to obtain the data. There are many uses for this data ranging from comparing nutrient intakes in populations, development of food based dietary guidelines, assessing dietary or meal patterns and risk assessment from foods. You should carefully consider what it is you would like to do with the data you are considering to ensure it is appropriate to answer your research question. For example, data collected using dietary recalls or food diaries is collected at an individual food level and usually has portion size information which produces detailed nutrient information. Data collected using food frequency questionnaires is collected at a food group level and may not contain portion size information, whilst nutrient information can still be obtained it is more of an estimate. If you are interested in a specific nutrient or meal patterns then data collected at an individual food level may be more appropriate. |
| **Consumer behaviour data domain** | |
|  | *Methods* |
| M5.1 | You reported that consumer behaviour data are available within this dataset. The consumer behaviour indices in the dietary intake dataset you have selected for reuse have been collected using qualitative methods. Data collected in this manner is usually not appropriate for merging with other datasets as the results are influenced by factors such as the researcher collecting the data and their training/experience; the original research question and topics explored; the method used to collect the data and the population group under study. The outputs from different qualitative datasets could be compared across different studies but the data itself is not usually appropriate for merging. |
| M5.2 | You reported that consumer behaviour data are available within this dataset. The consumer behaviour indices in the dietary intake dataset you have selected for reuse have been collected using an indirect approach. This includes data arising from cash registers or app tracking. It would be important for you to consider the completeness of this data and what the data was intended to capture; the population in which the data was collected in, and whether this data is appropriate for comparing to other populations. |
| M5.3 | You reported that the consumer behaviour data in this dataset was collected using a validated questionnaire. Data produced using validated questionnaires tends to be of better quality and is comparable with other datasets collected using the same questionnaire. It is important to consider whether the questionnaire has been validated in a similar population group/country to the study population in this dataset. |
| M5.4 | You reported that the consumer behaviour data in this dataset was collected using a questionnaire which has not been validated. Data produced using validated questionnaires tends to be of better quality and is comparable across datasets which have been collected using the same questionnaire. |
| M5.5 | You reported that the consumer behaviour data in this dataset was collected using a household budget survey. Consideration should be given to the length of time of data collection as data can vary across seasons. Consideration should also be given to the parameters of interest as eating-out is typically only assessed monetarily without specification of the types of foods or dishes consumed. Consider whether the data was collected in a representative or targeted sample (e.g., families or young people <30 years) as this may affect the data produced. |
| M5.6 | You reported that the consumer behaviour data in this dataset was collected using a method other than questionnaires or household budget surveys. You should consider whether this data was collected using a validated method, the population under study and the length of time of data collection as these factors can all influence the quality of the data produced. |
| **Demographic data domain** | |
|  | *Methods* |
| M6.1 | You reported that demographic data are available within this dataset. Demographic data includes factors such as age, sex, gender, ethnicity, nationality, religion, marital status, socioeconomic status, occupation, income, education and living situation. There are numerous ways in which demographic data is typically collected alongside dietary intake information including through phone or in-person interviews or else via questionnaires which can be self-administered or administered via an app or web-based survey. Regardless of the method chosen there are some common factors which should be considered by researchers when using this data. The terminology used throughout questionnaires may vary. When combining data, you should identify any variations in the terms used and consider whether this could change the interpretation or meaning of the question. For example, questions may use terms such as sex and gender, or ethnicity and nationality interchangeably however these are not collecting the same information. Furthermore, when reusing data or if combining different datasets you should consider the response options available to different questions. Questions with free text responses can provide more information when compared to closed responses (i.e., tick box). You should consider the response options available to each question and consider whether there is a response missing which could oversimplify the data produced (e.g., sex questions with only male/female responses may exclude those who identify as non-binary). Descriptive statistics are usually used to analyse demographic data and demographic information is best presented in tabular format to provide an overview of population of interest. Within dietary intake analysis, many demographic factors may be considered as confounding factors (e.g., socioeconomic status) which can be controlled for. |
| M6.2 | You reported that the demographic data within this dataset was collected using a method other than a questionnaire such as individual questions created de novo by the research team. Carefully consider the wording of these questions and the response options before merging them with other datasets to ensure that they are comparable. |
| M6.3 | You reported that the demographic data within this dataset has been collected using a validated questionnaire. Validated questionnaires tend to collect better quality and more accurate data. You should check whether the questionnaire was validated in a similar population to the one under study. |
| M6.4 | You reported that the demographic data within this dataset has been collected using a questionnaire that has not been validated. Demographic information in surveys is frequently collected using a questionnaire that the researcher has developed specifically for that study. Consider the terminology used in the questions and how this might impact the responses gathered (e.g., pre-defined answer options limits the ability to capture something outside of this) and the variable collected (many parameters which can be measuring the same thing). |
| M6.5 | You reported that the demographic data in this dataset was collected using a self-administered questionnaire where the participants were provided with instructions on how to accurately complete it. Provision of instructions for participants can improve the detail and quality of the information provided. |
| M6.6 | You reported that the demographic data in this dataset was collected using a self-administered questionnaire where the participants were not given prior instructions on how to accurately complete it. Provision of instructions for participants can improve the detail and quality of the information provided. |
| M6.7 | The demographic data within this dataset was collected using an interviewer-led questionnaire conducted by trained individuals. Evidence has demonstrated that when interviewers are trained the precision and accuracy of the data collected is improved. |
| M6.8 | The demographic data within this dataset was collected using an interviewer-led questionnaire conducted by individuals who were not trained. Evidence has demonstrated that when interviewers are trained the precision and accuracy of the data collected is improved. |
| M6.9 | You reported that the demographic data within this dataset has been collected using a questionnaire but you do not know whether this questionnaire was validated. Validated questionnaires tend to collect better quality and more accurate data. You should check whether the questionnaire has been validated and if it has whether the validation population is similar to the population in this dataset. |
| **Anthropometric data domain** | |
|  | *Methods* |
| M7.1 | You reported that anthropometric data are available within this dataset and that this data was self-reported by participants who were given prior instructions and/or trained in how to take the measurements. Although self-report bias in anthropometric measurements still exist, instructions and/or training can improve the accuracy of measures taken by participants. The researcher should also consider the population within the dataset. For example, older adults are more likely to report their height from their younger years and not account for possible height losses. Furthermore, participants who may have overweight or obesity are more likely to misreport their weights. |
| M7.2 | You reported that anthropometric data are available within this dataset and that this data was self-reported by participants who were not provided with instructions and/or trained in how to take the measurements. Although self-report bias in anthropometric measurements still exists, instructions and/or training can improve the accuracy of measures taken by participants. The researcher should also consider the population within the dataset. For example, older adults are more likely to report their height from their younger years and not account for possible height losses. Furthermore, participants who may have overweight or obesity are more likely to misreport their weights. |
| M7.3 | You reported that anthropometric data are available within this dataset and that this data was collected by a trained individual. Anthropometric data collected by trained researchers is more reliable and accurate than self-reported measures. The researcher should also consider whether there is a reference to the methodology used to collect these measures in the dataset release notes as use of published methodology increases the comparability and quality of the data produced. |
| M7.4 | You reported that anthropometric data are available within this dataset and that this data was collected by individuals who have not been trained. Anthropometric data collected by trained researchers is more consistent, reliable and accurate. The researcher should also consider whether there is a reference to the methodology used to collect these measures in the dataset release notes as use of published methodology increases the comparability and quality of the data produced. |
| **Lifestyle data domain** | |
|  | *Methods* |
| M8.1 | You reported that lifestyle data are available alongside this dietary intake dataset and that some of the lifestyle data within this dataset has been collected using a validated questionnaire. Lifestyle data can include information on factors such as activity level, sedentary behaviour, sleep, alcohol intake and smoking status. Data collected using validated questionnaires is of better quality and is easily comparable to data collected in other studies using the same questionnaire. The researcher should consider whether the questionnaire has been validated in a similar population to the one in this dataset. |
| M8.2 | You reported that lifestyle data are available alongside this dietary intake dataset and that some of the lifestyle data within this dataset has been collected using a questionnaire that has not been validated. Lifestyle data can include information on factors such as activity level, sedentary behaviour, sleep, alcohol intake and smoking status. Data collected using validated questionnaires is of better quality and is easily comparable to data collected in other studies using the same questionnaire. The researcher should carefully consider the terminology used in each question and the response options before merging them with other datasets. The terminology used within questions may vary which could change the interpretation or meaning of the question. For example, questions may use terms such as sex and gender, or ethnicity and nationality interchangeably however these are not collecting the same information. Furthermore, when reusing data or if combining different datasets you should consider the response options available to different questions. Questions with free text responses can provide more information when compared to closed responses (i.e., tick box). You should consider the response options available to each question and consider whether there is a response missing which could oversimplify the data produced (e.g., sex questions with only male/female responses may exclude those who identify as non-binary). |
| M8.3 | You reported that some of the lifestyle data within this dataset has been self-reported or collected using individual questions created by the original research team. Lifestyle data can include information on factors such as activity level, sedentary behaviour, sleep, alcohol intake and smoking status. The researcher should carefully consider the terminology used in each question and the response options before merging them with other datasets. The terminology used within questions may vary which could change the interpretation or meaning of the question. For example, questions may use terms such as sex and gender, or ethnicity and nationality interchangeably however these are not collecting the same information. Furthermore, when reusing data or if combining different datasets you should consider the response options available to different questions. Questions with free text responses can provide more information when compared to closed responses (i.e., tick box). You should consider the response options available to each question and consider whether there is a response missing which could oversimplify the data produced (e.g., sex questions with only male/female responses may exclude those who identify as non-binary). |
| M8.4 | You reported that some of the lifestyle data within this dataset has been collected using app tracking from wearables such as a pedometer or FitBit. You should learn more about the source of the data particularly if it's from a commercial app tracker (e.g., FitBit, Garmin) as the quality of data arising from commercial apps/wearable devices is very variable. |
| M8.5 | None of the lifestyle data collected within this dietary intake dataset is reported to have been collected using app tracking. |
| M8.6 | You reported that lifestyle data are available alongside this dietary intake dataset but that you do not know whether the questionnaire used to collect the lifestyle data has been validated. It would be important for you to find this out as data collected using validated questionnaires is of better quality and is easily comparable to data collected in other studies using the same questionnaire. Furthermore, if the questionnaire has been validated you should consider whether this was done in a similar population to the one in this dataset. |

**Supplementary Table 3: Specific suggestions for improvement in quality assessment tool**

| **Section of tool** | **Issue/Observation** |
| --- | --- |
| Landing page | - Provide a clear name or title of the tool |
| Introductory message | - Is fairly text heavy – is all information necessary? - “Metadata” may not be understood by all – rephrase or consider an explanation of the term - In nutrition, many researchers consider quality in terms of quality of dietary intake instead of quality of data. Important to make a clear differentiation - Why do researchers need to supply a research question? It is never referred to again during the assessment/report. If a research question is asked for, how much detail should users provide? This should be broken down more - Information on research question is not necessary as it is never referred to in the feedback messages |
| Functionality of the tool | - One participant would have preferred that the hints appear in text directly below the question rather than popping up beside question when hover over the “i” icon - Cannot move back through previous questions, the “back” button brings the user back to the beginning of the tool assessment - Panel of questions would be useful to provide an overview of what type of questions will/have been asked and to monitor progress of assessment - In the future could it be possible to assess multiple datasets at once - PDF report download broken in half - Headings should be given to each section of the tool so participants know what domain they are being asked about - Wrong message maybe shown for dietary under/over reporters – participant reported they did not assess but received a message as if they did - May be a bug for a lifestyle message – participants answered questions on lifestyle section of assessment but did not receive any feedback message (registered as “this message was skipped”) |
| Assessment – dietary intake data | - Q1 subjective/objective: needed to select a mixed methods approach but no option to do so - Biomarkers may be assessed but not for purpose of validation of subjective methods - Q1 subjective/objective: not understood well by all, some relied on the hint for clarity - Q4 method used: consider phrase “diet diary” – also required to “food diary record” - Q7 tool validation: slightly confusing. Could have been broken down into two sections (1=sample size, 2= population demographics) - Q11 type of diet diary: no icon appears on screen for this question - Q11 type of diet diary: what is meant by novel and why is paper based not novel? - Q11 type of diet diary: what is meant by training? Does it mean guidance or formal training? - Although use of food composition data databases may be referenced, the data used may not be as recently updated as the composition databases - The term sensitivity analysis was not known by all participants and relevance of this was queried (should researchers not do this themselves when analysing the data?) - Food coding system – make question more specific on whether they use a local or international food coding system - Same questions for food diaries are not all necessary for FFQs |
| Assessment – Other data | - Some questions (lifestyle in particular was mentioned) are very general and difficult to make specific to research topic - Be clearer on what is included by consumer behaviour data – provide a definition or some context at the beginning |
| Feedback Messages | - Information although relevant could be presented in an easier way - Are you trying to provide guidance or assess quality of datasets? Needs to be clear and all text in messages therefore may not be necessary - Provide a summary overview at the end about the quality of the dataset or at least highlight main weaknesses that researchers should be aware of. A table summary or a checkbox list were suggested - Dietary intake message appears twice, keep all messages combined at the end - Add a section where useful links, resources and relevant literature could be listed for users - Could be useful to separate the report into two section 1=specific to the answers provided/users dataset and 2=general advice for maintain data quality using nutrition data - Data analysis - link to previous literature and discuss recommended approaches |
| Typos/errors | - Demographic message “ppresented” |
| Additional questions/ comments | - Ask about representativeness of the sample (sample size, information on age range/ population in general) - How were under reporters identified? - Examining short term of long term intakes? Number of assessments which were completed, were they on consecutive or non-consecutive days? - Was anthropometric data collected fasted/unfasted, were measurements validated if taken by multiple researchers? - Is it good or bad that no data was collected using app tracking – comment within report. - Consider more novel assessment methods (e.g. glucose monitoring) - How was missing data dealt with? - Consider seasonal variation of intake? - Measurement of eating habits (food purchases, eating setting, cooking skills etc) |

**A.**
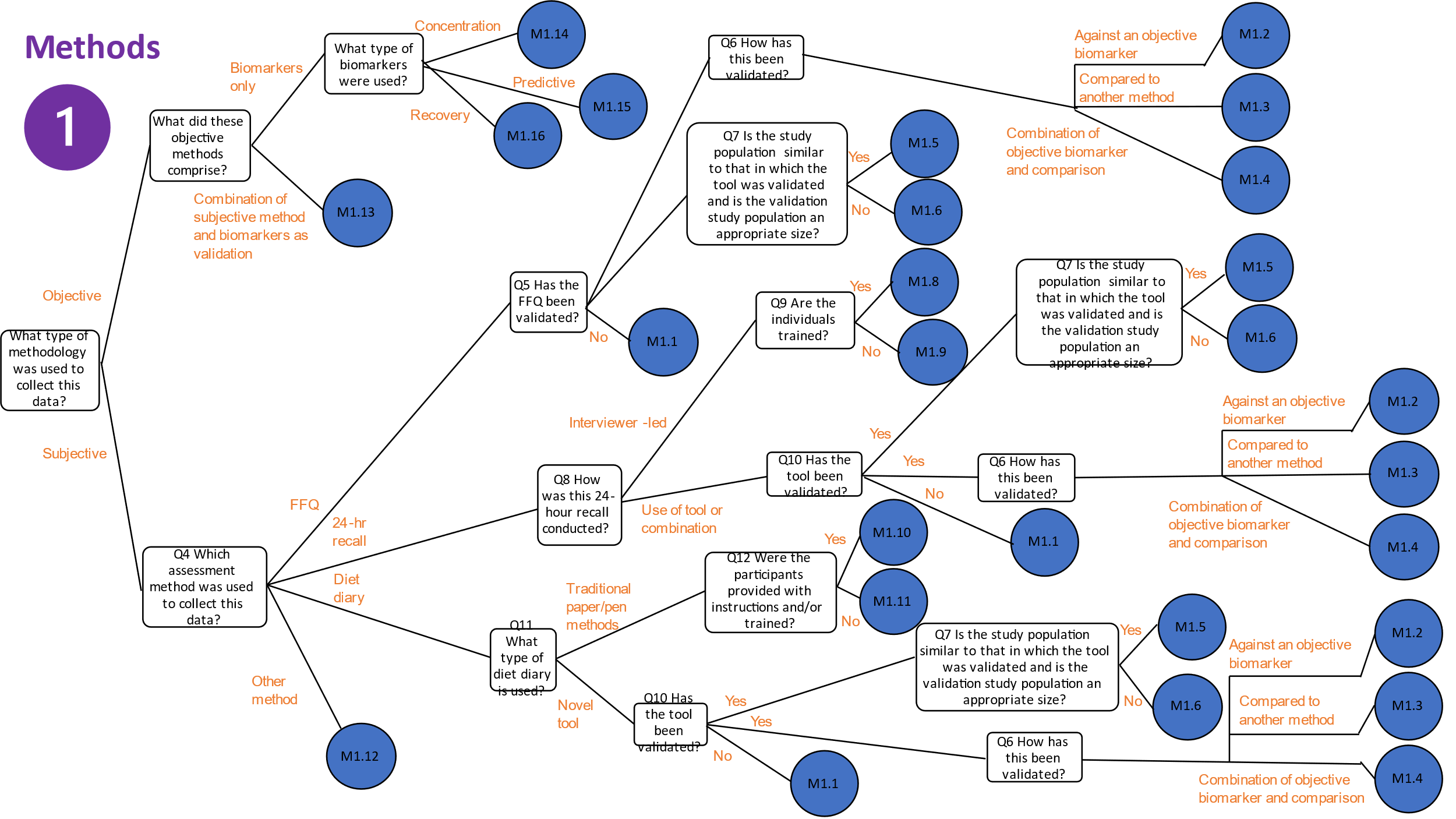


**B.**
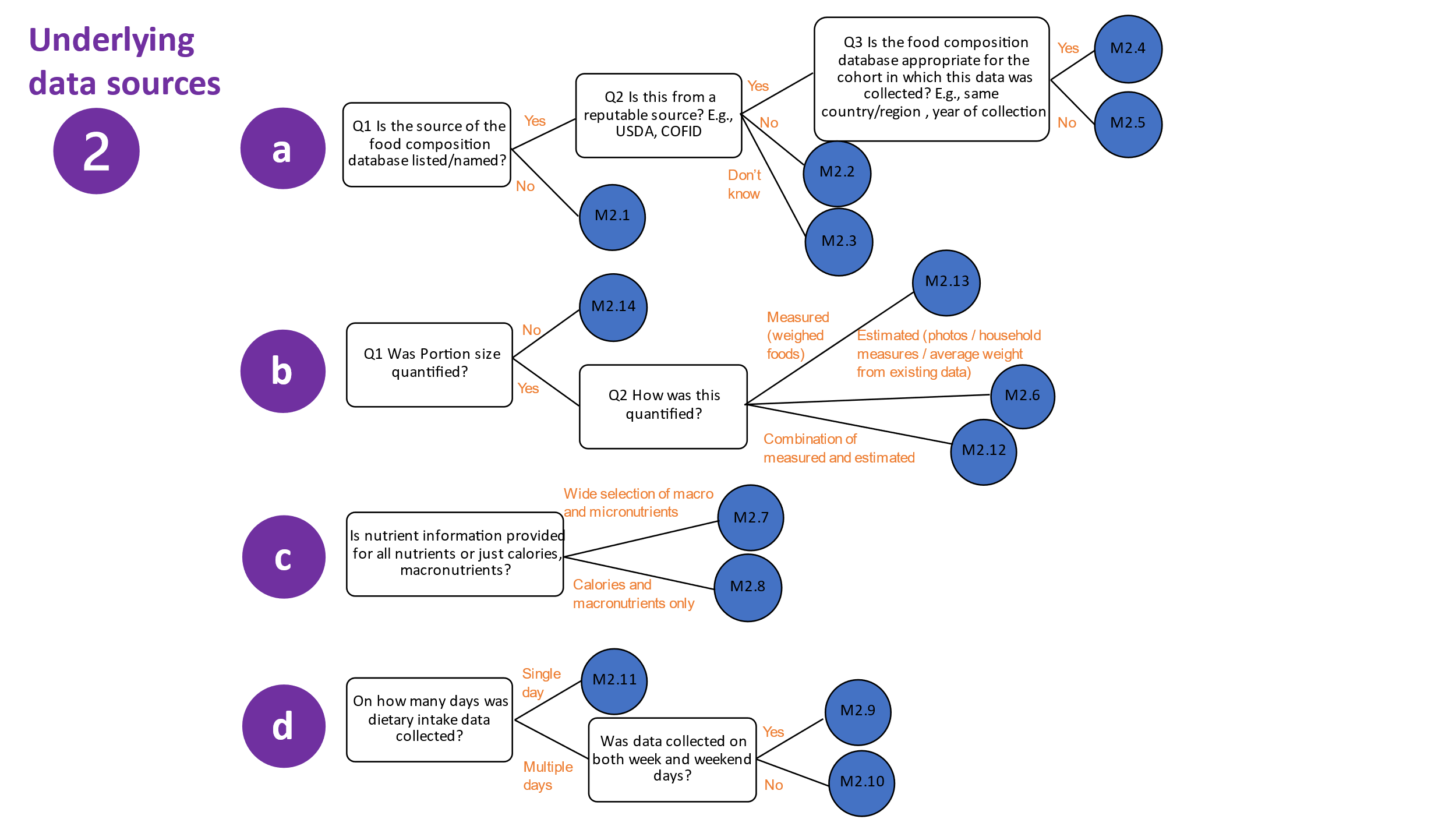


**C.**
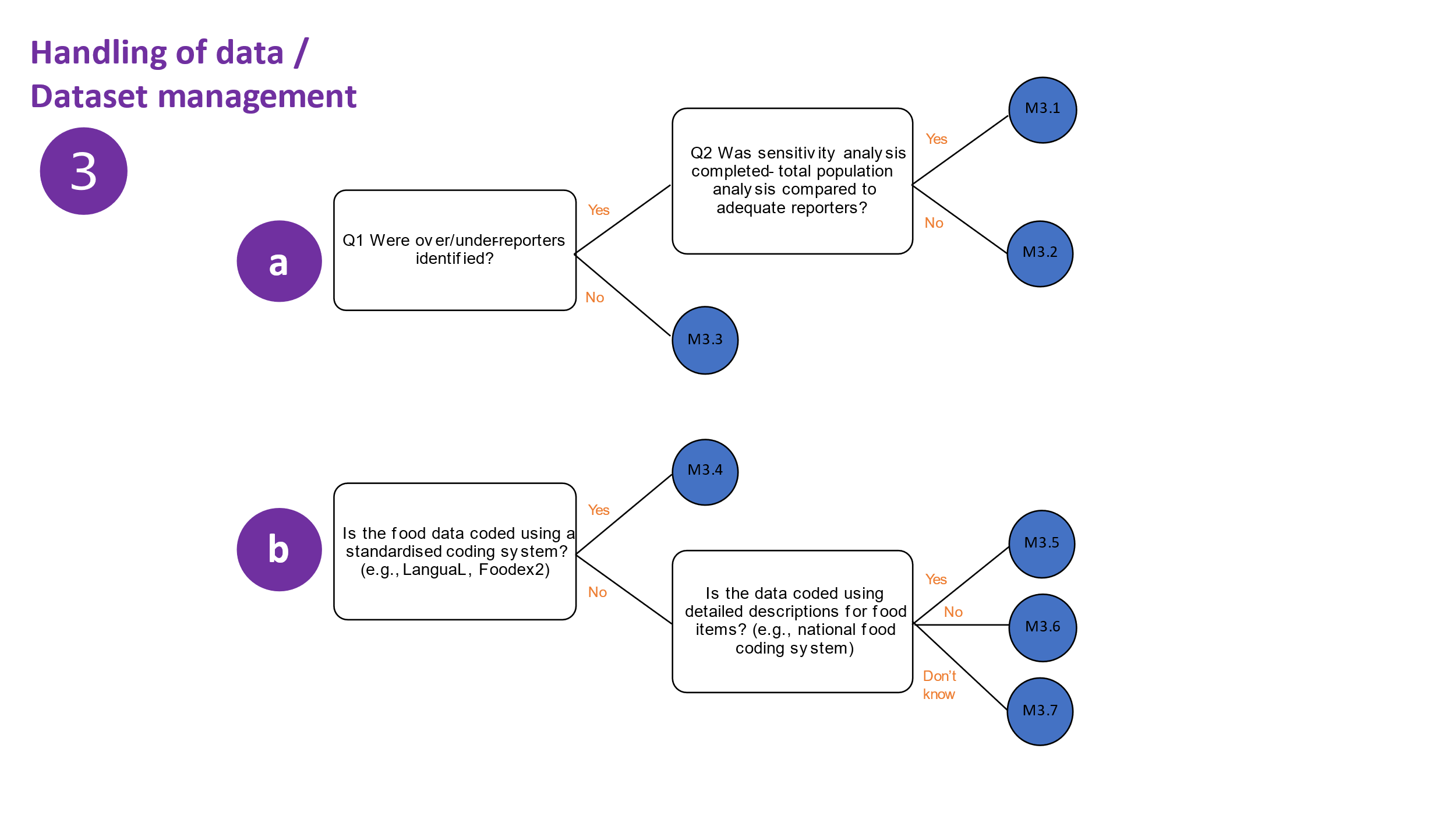


**Supplementary Figure 1a-c. Structure of branches of flowcharts in dietary intake data domain**

A. Methods branch of dietary intake datasets flowchart; B. Underlying data sources branches of dietary intake datasets flowcharts; C. Handling of data branches of dietary intake datasets flowcharts. Questions are presented in rectangular, clear boxes; response options are presented in orange text, numbers depicted in blue circles represent the end of the flow and that a message will be shown.
